# Machine Learning Approaches for Predicting Fatty Acid Classes in Popular US Snacks Using NHANES Data

**DOI:** 10.3390/nu15153310

**Published:** 2023-07-26

**Authors:** Christabel Y. E. Tachie, Daniel Obiri-Ananey, Nii Adjetey Tawiah, Nii Attoh-Okine, Alberta N. A. Aryee

**Affiliations:** 1 Food Science and Biotechnology Program, Department of Human Ecology, College Agriculture, Science and Technology, Delaware State University, 1200 N DuPont Highway, Dover, DE 19901, USA; 2 Department of Computational Data Science and Engineering, North Carolina Agricultural and Technical State University, 1601 E Market St, Greensboro, NC 27411, USA; 3 College of Humanities, Education and Social Sciences, Delaware State University, 1200 N DuPont Highway, Dover, DE 19901, USA; 4 A. James Clark School of Engineering, Civil and Environmental Engineering, University of Maryland, 4298 Campus Dr., College Park, MD 20742, USA

**Keywords:** NHANES, popular snacks, energy-dense foods, fatty acid classes, machine learning

## Abstract

In the US, people frequently snack between meals, consuming calorie-dense foods including baked goods (cakes), sweets, and desserts (ice cream) high in lipids, salt, and sugar. Monounsaturated fatty acid (MUFA) and polyunsaturated fatty acid (PUFA) are reasonably healthy; however, excessive consumption of food high in saturated fatty acid (SFA) has been related to an elevated risk of cardiovascular diseases. The National Health and Nutrition Survey (NHANES) uses a 24 h recall to collect information on people’s food habits in the US. The complexity of the NHANES data necessitates using machine learning (ML) methods, a branch of data science that uses algorithms to collect large, unstructured, and structured data sets and identify correlations between the data variables. This study focused on determining the ability of ML regression models including artificial neural networks (ANNs), decision trees (DTs), k-nearest neighbors (KNNs), and support vector machines (SVMs) to assess the variability in total fat content concerning the classes (SFA, MUFA, and PUFA) of US-consumed snacks between 2017 and 2018. KNNs and DTs predicted SFA, MUFA, and PUFA with mean squared error (MSE) of 0.707, 0.489, 0.612, and 1.172, 0.846, 0.738, respectively. SVMs failed to predict the fatty acids accurately; however, ANNs performed satisfactorily. Using ensemble methods, DTs (10.635, 5.120, 7.075) showed higher error values for MSE than linear regression (LiR) (9.086, 3.698, 5.820) for SFA, MUFA, and PUFA prediction, respectively. R^2^ score ranged between −0.541 to 0.983 and 0.390 to 0.751 for models one and two, respectively. Extreme gradient boost (XGR), Light gradient boost (LightGBM), and random forest (RF) performed better than LiR, with RF having the lowest score for MSE in predicting all the fatty acid classes.

## 1. Introduction

Most often energy-dense snacks are consumed in between meals. Frequently, they are low in micronutrients but high in calories, lipids, salt, or sugar [1,2]. Hunger, availability, food culture, boredom, and interruption are significant drivers of snacking [2]. In addition, due to urbanization, most consumers are desk-bound and inactive and do not expend energy from energy-dense foods. This contributes to obesity and other diet-related health problems and has become a growing concern worldwide [3]. In addition, consuming a diet low in sodium and saturated fatty acids (SFAs) helps in maintaining healthy blood pressure and low blood cholesterol level, respectively [4]. The high incidence of essential hypertension has led to increased efforts to reduce sodium and certain classes of fatty acids (FAs) in the US food and snack supply. This involves accurately assessing and monitoring snacks’ lipid, sodium, and related nutrient content.

Fatty acids (FAs), the building block of triglycerides, are the predominant components of fats and oils, including three main classes—saturated, monounsaturated, and polyunsaturated fatty acids. Although dietary lipids benefit the human body by increasing the absorption of fat-soluble vitamins, providing concentrated energy, insulating to conserve heat, serving as a lubricant for body parts [5], and adding flavor to food, the excessive consumption of certain classes of FA has been linked to increased risk of obesity, liver disease, and elevates serum cholesterol levels, which may lead to cardiovascular diseases [5,6]. Most of their intake is estimated to be contributed by commercially processed foods, including snacks and restaurant foods [7,8] with varying FA classes. Unlike saturated fats, unsaturated oils such as olive are healthy and are less likely to cause adverse health effects. SFA is a more significant contributor to obesity than MUFA and PUFA [9]. In addition, these two FA classes are readily absorbed as compared to a slower absorption of SFA. The daily recommended intake for SFA is less than 10% of calories starting at age two [10]. Other health disorders associated with obesity are musculoskeletal disorders and some cancers (liver, breast, and colon) [11]. Currently, the effective practice for primary prevention of type 2 diabetes in adults includes lowering the consumption of certain types of dietary fat and increasing fiber-rich and whole-grain foods [12]. Interventions in nutrition are geared toward changing dietary FA to minimize cardiovascular diseases [13]. To this end, the Center for Disease Control and Prevention (CDC) conducts an annual nutritional survey in the US that seeks to establish the relationship between diet and diseases among the population [14]. Information obtained is stored in databases such as the NHANES and available for researchers to study. Food and nutrition-related studies, such as diet and its association with health, produce a wealth of data [15]. However, classical statistics is not adequately equipped to develop prediction algorithms to analyze large datasets, mostly non-linear with complex interactions, and many predictor variables that need to be combined to generate predictive models [12]. The need for rapid analysis of large datasets to develop dietary recommendations and to elevate businesses has increased the use of data science in food science [16].

Data science involves using mathematical techniques and algorithms to teach or train computers to develop models with high accuracy and predictive abilities to gain insight into data [17,18]. Artificial intelligence (AI), a subgroup of data science, uses computer simulations of human intelligence to carry out predetermined tasks independently or partially autonomously [19] and has also found application in the food industry. For instance, it is used by multinational food and beverage corporations to facilitate subtle nudges [20]. The analysis of 12 global food companies’ annual reports from 2015 to 2019, including Nestle, Danone, McDonald’s, and Coca-Cola, showed how artificial intelligence (AI) technologies like facial recognition, social listening, virtual reality, recommender systems, and robots influenced consumer decisions. These industries used these methods to recommend new items online through advertisements, track social media trends, respond to customer feedback and ideas, and use cookies on numerous websites to determine the age range of their consumers to inform decision-making [20]. ML, a subset of AI, has improved hardware and software systems capable of rapidly deriving useful information from data and utilizing that information to self-learn and subsequently make reasonable classifications and predictions [21]. Although ML can extract meaningful information from data, it must be combined with already existing data obtained from previous experimental analysis or a stored database. Data science methods demand large and more informative datasets, which can only be acquired when the measuring parameters are adequately adjusted [18].

Based on the characteristics of the data being learned and the expected results, ML can be categorized into supervised, unsupervised, and reinforcement learning [19,21,22]. Supervised ML models can be applied for classification or regression when the dependent variable is nominal or quantitative [23], while the subdivision of unsupervised ML models involves dimension reduction and data segregation into clusters [24]. The selection of ML algorithms depends on the research problem, the number of variables, and the model that best suits it [25]. For example, ML algorithms have been used to authenticate food using specific markers analyzed in nutritional studies to predict the association between obesity and demographics (age, body mass index, gender) [26], to distinguish between products based on their species, geographical origin [27], and production method [28], and to predict the quality of plant-based foods [29].

ANN, KNN, DT, and SVM are examples of ML algorithms used to study relationships and patterns, reveal hidden trends, and draw necessary conclusions from the data [30]. ANN consists of an input, hidden, and output layer [31]. The three layers are collectively called multi-layer perceptron (MLP) and are used to learn, generalize, and extract information from data [32]. ANN model predicts better when data are non-linear and complex, irrespective of how the data are distributed [33]. It is also well suited for data whose variances are not constant by learning confidential and detailed information due to their modeling architecture [33]. ANN and SVM are the two most popular algorithms because they are adaptable to processing large and complicated datasets with greater accuracy than other supervised ML algorithms [34], though the selection largely depends on data characteristics and modeling tasks. The KNN algorithm is a simple supervised model used for classification and regression. It uses the nearest data points to make predictions for unseen datasets, and its performance depends on the distance metric and k-values [35].

Most databases are restricted to nutrient information acquired via laborious chemical analysis, recipe calculations, volume estimation, and food imputation. These procedures are time-consuming and require rapid approaches to determine nutrient content. ML tools may offer a cost-effective method to predict a food’s nutritional value, including snacks. This present study is aimed at evaluating eight ML algorithms for quantitative prediction of SFA, MUFA, and PUFA content in popular snacks consumed in the US using NHANES data from 2017–2018.

## 2. Data Source

The NHANES is a nationally representative, cross-sectional survey designed to monitor the health and nutritional status of the civilian, noninstitutionalized U.S. population and is conducted by the CDC’s National Center for Health Statistics [36]. NHANES data are collected through interviews and standardized examinations [37]. Trained interviewers use the 5-step computerized United States Department of Agriculture (USDA) Automated Multiple-Pass Method (AMPM) to collect 24-h recalls [38]. What We Eat in America (WWEIA) is the dietary interview component of the NHANES data. The snack categories: savory snacks, crackers, snack/meal bars, sweet bakery products, candies, and other desserts used in this study were specified by WWEIA. The snack types consist of ‘ice cream and frozen dairy desserts’ (5.32%), ‘pudding’ (3.38%), ‘cakes and pies’ (44.86%), ‘tortilla, corn, other chips’ (2.45%), ‘doughnuts, sweet rolls, pastries’ (11.83%), ‘crackers, excludes saltines’ (3.84%), ‘cookies and brownies’ (6.68%), ‘pretzels/snack mix’ (4.35%), ‘cereal and nutrition bars’ (2.45%), ‘popcorn’ (3.25%), ‘gelatin, ices, sorbets’ (1.18%), ‘potato chips’ (1.69%), ‘candy not containing chocolate’ (4.56%), and ‘candy containing chocolate’ (4.14%). Dietary data including SFA, MUFA, and PUFA were obtained from the Food and Nutrient Database for Dietary Studies (FNNDS) from the 2017–2018 NHANES data (https://wwwn.cdc.gov/nchs/nhanes/search/datapage.aspx?Component=Dietary&Cycle=2017-2018, accessed on 25 February 2022) using a 24-h recall of the snacks usually consumed by people in the US. This study included data such as total fat content, ingredient weight, snack type, and calories of 2367 popular snacks consumed in the US to train algorithms to predict the amounts of FA classes (SFA, MUFA, and PUFA) in snacks.

## 3. Modeling

The Orange software, version 3.31, used in this study has tools known as widgets. The data were uploaded into the file widget, then the parameters (target and feature) were selected from the various categories in the data. The independent variables included energy, snack type, ingredient weights, total fat content, method of preparation, major nutrient, and ingredient description while the dependent variables were SFA, MUFA, or PUFA. The default pre-processor, which is included in the models, was used. Pre-processing activities included replacing all missing values (0.1%) with averages, transforming categorical variables into numbers, and scaling by standardization. Standardization was selected because it is less sensitive to outliers, preserves original data distribution, and was compatible with the selected algorithms such as KNN and ANN for stability during training. The data were then split into 70% and 30% for training and testing, respectively. The ML algorithms, KNN, ANN, SVM, and DT were used to train and test the data. The rectified linear activation unit (ReLU) function with 200 iterations and 100 neurons in the hidden layer was used for the ANN model. The radial basis function (RBF) with 100 iterations was used for SVM. For the KNN, distance weight was selected with Euclidean metrics. These combinations resulted in the best performance for each model. Finally, the model output was evaluated based on the following metrics: root means squared error (RMSE), mean absolute error (MAE), mean square error (MSE), and coefficient of determination (R^2^). Python software version 3.10.6 was used to analyze the same data to create a second model using 500 selected popular snacks from various categories. The 500 selected snacks were from the categories with relatively high amounts of saturated fatty acid. Using different sets of data provides an additional “cushion” in addressing model uncertainty and the stability of the models.

After importing libraries, including pandas (version 1.3.5), seaborn (version 0.11.2), and NumPy (version 1.21.6), the data were uploaded and pre-processed. Pre-processing involved missing value treatment, variable transformation, and reduction. A summary of the data, including shape, range, mean, first, second, and third quartiles, was obtained. One row with missing values was deleted. Then, a box and whisker plot was generated to determine the data distribution. The features included snack type, flavor type (chocolate, vanilla, and fruits), major nutrients (proteins, carbohydrates, and fats) and primary methods (fermentation: yogurt, baking: cakes and doughnuts, frying: chips and popcorn) energy, total fat, SFA, MUFA, and PUFA. Processing methods, such as frying, for instance, have been reported to contribute to the fatty acid content in foods. Flavors such as chocolate snacks contribute to the overall fatty acid content. Feature transformation was done by replacing the non-numerical categories (flavor type, major nutrient, snack type, and preparatory method) with unique integer values using the LabelEncoder function since the variables had no order or hierarchy, and feature selection was made using the permutation_importance function from the scikit-learn library to compute the most significant variable contributing to the generation of the final model. The less essential features, which did not contribute to the modeling (food code, category description, sequence number) were dropped. A heat map was generated to find the correlation between the dependent and independent variables. K-fold cross-validation was used to train and test the datasets using k-folds of 5 and a random state of 100. The ML algorithms (extreme gradient boost regressor (XGR)), DT, light gradient boost model (LightGBM), and RF were used for predicting SFA, MUFA, and PUFA contents in snacks, using the total fat content. The models were also evaluated with the metrics mentioned above.

**Figure 1:**
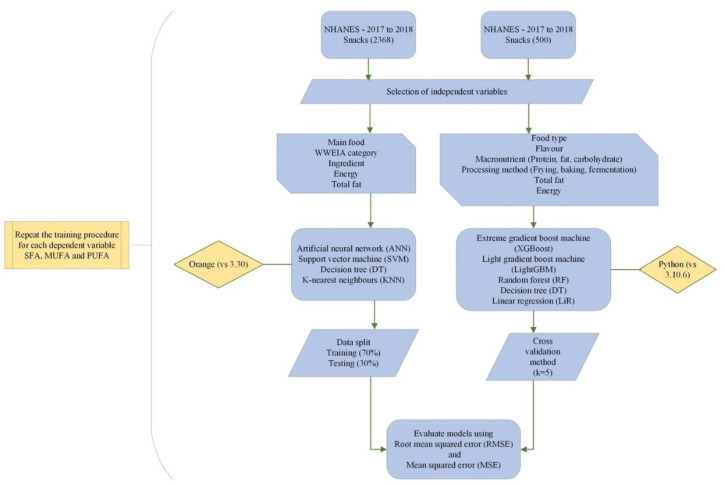
Workflow for fatty acid class prediction for models 1 and 2.

Other ML models include random forest (RF), partial least squares (PLS), partial least squares discriminant analysis (PLS-DA), principal component analysis (PCA), linear regression (LiR), logistic regression (LR), and KNN. PLS is a dimension reduction algorithm that minimizes multicollinearity in a dataset and can be used for multivariate outcomes [39]. Multicollinearity occurs if the independent variables in the dataset correlate with each other. It results in errors when building models. PLS regression is used when the dependent variable is continuous, and PLS-DA is applied for categorical dependent variables [39]. PCA is an unsupervised ML algorithm that recognizes patterns and is a feature extraction tool. It also gives knowledge on the ability of the data to be used for model creation through correlation [40] by restructuring data into principal components (PCs) based on the variance. If the sum of the first few components (PC1 and PC2) has a 95% variance or more, the data are regarded as dimensionally reduced with minimal loss of information [41]. Since it distinctively separates similar variables into groups, when these groups overlap, classification accuracy is minimized [27]. Linear regression mainly predicts the relationship between a continuous dependent variable (quantitative) and an independent variable by fitting a regression line to reduce the residual error [42]. Its modeling performance is enhanced with datasets devoid of multicollinearity [39]. Although regularization can decrease overfitting in LR, the major demerit is its ability to simplify practical problems [43]. The experimental workflow for the modeling process is illustrated in Figure 1.

## 4. Results and Discussion

The first modeling was done using the entire snack dataset. The results indicated that popular snacks sold in the US contain all three FA classes with 32.62% SFA, 34.84% MUFA, and 25.57% PUFA. Most of the snacks consumed in India contain all three FA classes [44]. Research has shown that a high-fat diet, especially one high in SFAs, increases the risk of diseases such as obesity, diabetes mellitus, cancers, and cardiovascular diseases. Frozen fruit juice bars in the ‘gelatin, ice, and sorbet’ category had between 20–60 kcal and 0–0.01 g total fat compared to chocolate-covered and nut candies with about 500–590 kcal and the highest total fat content ranging between 30–43.47 g. Chocolate-covered and nut candies had the worst composition. Therefore, the ML approach was used as a high computational technique to extract essential features from datasets used in this study (NHANES 2017–2018) to rapidly predict the type of FA in a food product based on the total fat content.

All the models for all the FA classes (Table 1) showed a slight difference between the values obtained for the training and testing dataset metrics. Low error rates in models depict high prediction abilities (Table 1). The error (MSE, RMSE, and MAE) margin obtained for the SVM model was very high in all the predictions of the FA classes (SFA > PUFA > MUFA). KNN consistently outperforms DT, SVM, and ANN across various performance metrics for all FA classes. It achieves lower errors (MSE, RMSE, MAE) and higher R^2^ scores, indicating better accuracy and ability to explain the variance in the data (Table 1). ANN shows moderate performance, but it falls short compared to KNN in terms of accuracy and ability to explain the variance. To enhance the prediction performance of the ANN algorithm, the model must be optimized using algorithms, such as gradient descent, to test many variables and select the one with the least error. For this study, the ANN model was not optimized, which could be a reason for its satisfactory performance.

In this study, KNN and DT predict values close to the values of interest [33]. Feature reduction is one characteristic that improves data. For instance, models such as KNN perform well when the number of columns/features are reduced [45]. In this study, after eliminating variables such as food code, sequence number, and ingredient description, which were not needed for prediction, the number of features was reduced and helped to improve the data. This may explain why the KNN was the most suitable model for this dataset, given its consistent and superior performance. KNN also uses all the instances in the training dataset to make predictions when a new dataset (test data) is given [35] and is a straightforward algorithm that makes no assumptions about datasets. It only stores the information from the trained data and uses the similarities between the trained data and new data to predict values [35].

SVM was unsuitable for this work compared to the other algorithms, as indicated by higher MSE, RMSE, and MAE values for all fatty acids in training and testing. The R^2^ scores for SVM are negative or near zero, suggesting that the model does not sufficiently capture the underlying patterns and explain the data variance. It often overfits the model, minimizing its predictive power, which could be a reason for its low scores in this study [30]. SVM’s performance might be affected by the choice of hyperparameters, or the linear nature of the model compared to the other models. Additionally, it is most suited for binary classification problems [46]. SVM is mainly used as a classifier and not as a regression model. This could influence its inability to predict the FA, as the data used in this study were continuous. These techniques can help generate ML models suitable for predicting different nutrient content in foods.

Table 2 summarizes the quantitative dependent and independent variables in the dataset. The NHANES data are non-linear, and it can be inferred from the summary that the dataset is skewed. This is because a large part of the data falls within the third quartile while the first quartile is occupied by a few datasets across all the FA classes.

Building a potent model involves problem formulation or data acquisition, tidying data, pre-processing, train-test split or cross-validation, model building, validation, prediction, and evaluation of model accuracy. For example, k-fold cross-validation is an evaluation technique that avoids overfitting by splitting different datasets into a specified number of k-fold for training and testing to accurately assess a model’s performance [47]. Creating a second model using selected snacks and ensemble methods yielded slightly different results.

**Figure 2:**
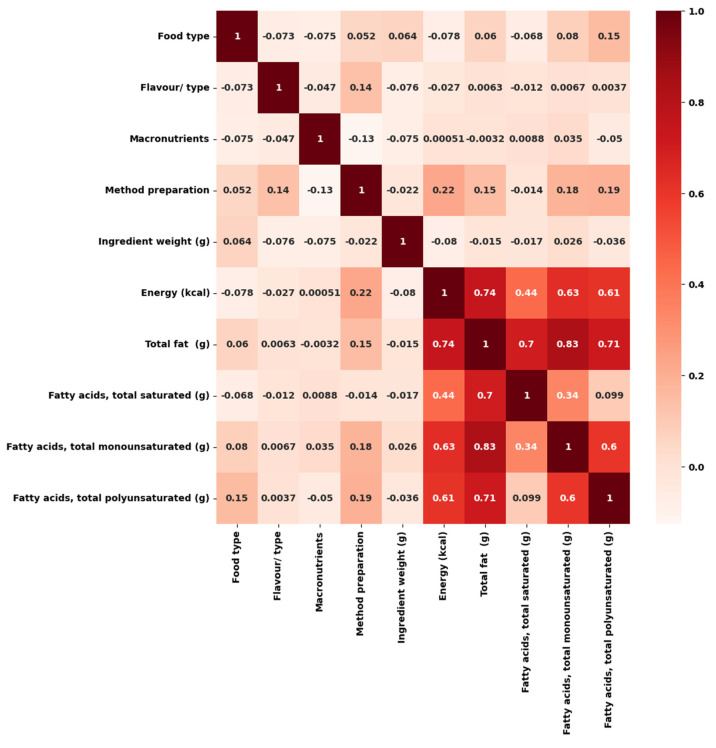
Correlation matrix of variables.

The magnitude of the correlation coefficient (r) reveals how strongly an association exists. The correlation matrix also aids in feature selection to detect multicollinearity among independent variables, which affects the final model’s performance (Figure 2) [48]. Two independent variables should not have a high correlation. The correlation matrix (Figure 2) showed a strong positive correlation among the independent variable, total fat, and dependent variables MUFA, PUFA, and SFA, with the highest correlation (0.83) between the total fat and MUFA. A robust positive correlation from 0.70 and above means that between two variables, one increases with a corresponding increase in the other [49], hence MUFA, PUFA (0.70), and SFA (0.71) increased in the snacks as the total fat content increased, with MUFA having the highest percentage increase.

**Figure 3:**
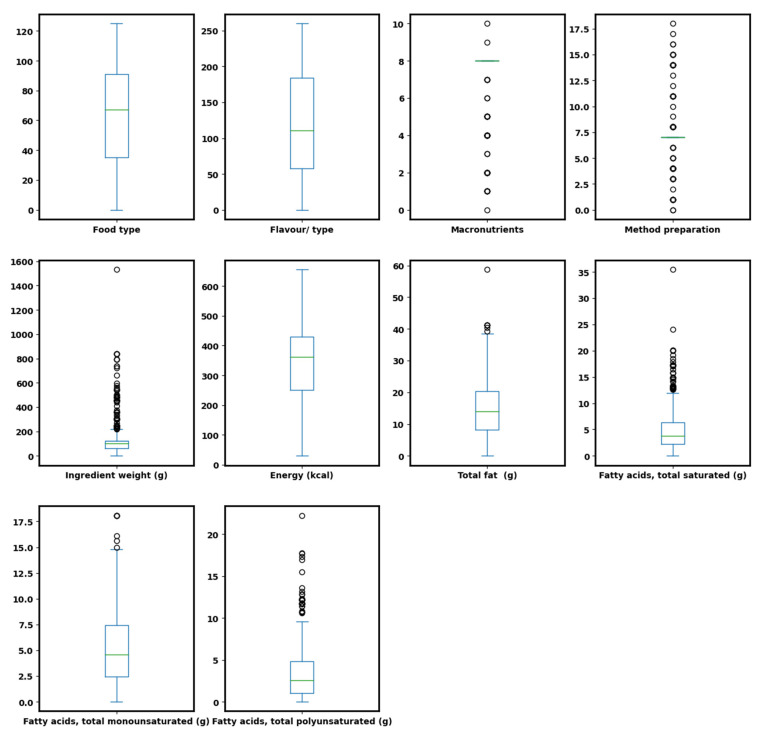
Boxplot of NHANES data used showing variable distribution.

Outliers are easily visualized using box and whisker plots, as illustrated (Figure 3), facilitating their treatment in the dataset. Points that lie outside the interquartile range of the dataset are outliers. For example, from Figure 3, features such as SFA, major nutrients, and ingredient weight had outliers. Ensemble and gradient boosting methods, including RF, XGR, and LightGBM, can analyze data (both linear and non-linear) without linear model assumptions to get accurate results and treat outliers [50]. Therefore, treatment of the outliers was not necessary. Linear models, such as LiR, on the other hand, make assumptions where data variables, in which multicollinearity and outliers exist, might result in lower algorithm performance. Therefore, such models may not be suitable for NHANES data.

**Figure 4:**
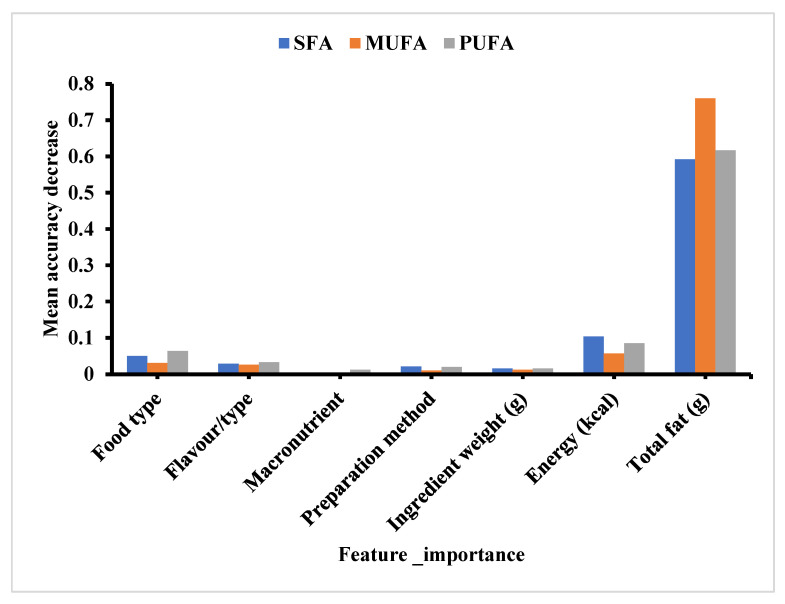
Feature importances that contribute to the final prediction of fatty acid classes in popular US snacks.

Model 2’s parameter tuning, and evaluation of essential attributes allowed it to find the ideal configuration to provide the highest accuracy [51]. The contribution of the independent variables in the various predictions is illustrated (Figure 4). The feature that contributed most to the accurate prediction of all three FA classes was total fat. Variations in “total fat” in food are closely related to changes in SFA, MUFA, and PUFA levels. As a result, the models perceived “total fat” as a strong predictor of these specific FA classes. Therefore, using significant parameters as inputs that directly affect the targets suggested provides robust ML models [52]. The prediction scores also differed depending on the fatty acid class (Table 1 and Table 2). This may be attributed to the data distribution, with MUFA achieving the best scores across all the models. As variables whose distribution is closer to normal, MUFA had the highest R^2^ values compared to the other FA classes (Table 1 and Table 3), with corresponding lower MSE, RMSE, and MAE rates [29,53].

The model performance is summarized in Table 3. Ensemble techniques act as a “black box” that uses numerous features from a dataset and mixed models, typically tree models, to enhance them [54]. LiR was used as a baseline model for comparison. LiR had the highest scores for MAE in all FA classes; however, its error rate was slightly lower than DT for RMSE and MSE. RF showed the least error scores for RMSE, MSE, and MAE in predicting all content of the individual FA classes and was, therefore, the best prediction model. Higher R^2^ values are also associated with decreased error scores, as seen in the MAE, RMSE, and MSE in all the fatty acid models, which indicates that these models were better at prediction. R^2^ ranges between 0–1, with 1 being a perfect fit and capturing a significant portion of data variance and 0 meaning no relationship between the predicted and actual value. The R^2^ scores ranged from 0.695 to 0.724, 0.390 to 0.514, 0.635 to 0.722, 0.503 to 0.622, and 0.686 to 0.751 in XGR, DT, LightGBM, LiR, and RF, respectively, for the FA classes. XGR, LightGBM, and RF exhibited higher R^2^ values, indicating that these have a better ability to explain the variance in the data and accurately capture trends between input features and the target variable. The success of XGR, LightGBM, and RF can be attributed to their ability to grasp complex relationships and handle non-linear patterns in the data. RF involves an ensemble of decision trees, which reduce overfitting [30]. Additionally, XGR and LightGBM XGR’s performance can be attributed to the power of gradient boosting algorithms, which iteratively improve the model’s predictions by combining weak individual models in an ensemble to provide faster and more accurate predictions [54,55]. DT and LiR had low R^2^, meaning that the underlying patterns in the data were not captured effectively by these two models, which resulted in larger error rates. DT and LiR, being simpler models, may struggle to capture the complexities and non-linearities in the data, leading to inferior performance.

RF showed the highest R^2^ (Table 3) in predicting all the fatty acids, except in PUFA, where the R^2^ of XGR was slightly higher (Table 3).

## 5. Study Limitation

The NHANES data on snacks from 2017 to 2018 was collected on different days across the country and most manufacturers improve or develop new products each day. The data do not fully represent all snacks consumed in the US.

## 6. Conclusions

Machine learning techniques can model complex non-linear datasets by incorporating interactions between sparse matrices and nutrition survey data with content variables like fatty acid classes and snacks to find non-linear relationships between the outcomes that conventional regression models might miss. Based on the raw data of total fat content, KNN and DT could predict with significant accuracy the classes of fatty acids in popular snacks. In the second model, RF followed by XGR most accurately predicted the fatty acid classes, while DT was the least effective. However, it is important to note that choosing the best model depends on various factors, such as the specific problem, dataset size, interpretability requirements, and computational constraints. Faster determination of these nutrients in foods through these models could promote intervention strategies by regulatory bodies to generate new or combined ingredients which can minimize calorie intake from snacks by consumers. It will increase awareness of the healthiness of different foods, and cater to consumers’ demand for personalized nutrition. Deep learning concepts could be developed for other foods that rely on tedious analytical/instrumentation methods to save time and minimize waste. Given sufficient data, ML algorithms could serve as a faster and more cost-effective means of predicting nutrient content in food.

## Figures and Tables

**Table 1:** Model comparison for predicted fatty acid classes in popular snacks in the US.

Model	Parameter	MSE			RMSE			MAE			R^2^		
		SFA	MUFA	PUFA	SFA	MUFA	PUFA	SFA	MUFA	PUFA	SFA	MUFA	PUFA
KNN	Training	0.616	0.229	0.505	0.785	0.479	0.711	0.190	0.135	0.175	0.966	0.982	0.959
	Testing	0.500	0.239	0.357	0.707	0.489	0.612	0.194	0.139	0.179	0.974	0.983	0.966
DT	Training	1.203	0.512	0.795	1.097	0.715	0.892	0.376	0.257	0.298	0.934	0.960	0.935
	Testing	1.137	0.715	0.544	1.172	0.846	0.738	0.417	0.301	0.290	0.927	0.948	0.940
SVM	Training	20.760	10.173	18.764	4.556	3.190	4.332	3.889	2.579	3.880	−0.142	0.212	−0.540
	Testing	21.023	9.986	18.125	4.585	3.160	4.257	3.925	2.528	3.835	−0.114	0.276	−0.660
ANN	Training	9.083	3.511	5.859	3.014	1.874	2.421	2.058	0.212	1.620	0.500	0.728	0.519
	Testing	9.227	3.757	5.280	3.038	1.938	2.298	1.993	1.888	1.536	0.511	0.728	0.516

Mean squared error (MSE), root mean squared error (RMSE), mean absolute error (MAE), and coefficient of determination (R^2^) of K-nearest neighbor (KNN) for decision tree (DT), support vector machine (SVM), artificial neural network (ANN) models for predicting the saturated fatty acid (SFA), monounsaturated fatty acid (MUFA), and polyunsaturated fatty acid (PUFA) content.

**Table 2:** Descriptive statistics for quantitative variables summarizing data range, quartiles, and the average content of energy and each fatty acid class.

Parameter	Energy (kcal)	Total Fat (g)	SFA (g)	MUFA (g)	PUFA (g)
Count	499	499	499	499	499
Mean	343.909	14.829	5.062	5.100	3.601
Std	113.318	8.927	4.367	3.430	3.495
Minimum	30.000	0.000	0.000	0.000	0.000
25% (1st quartile)	251.500	8.210	2.277	1.431	1.040
50% (2nd quartile)	362.000	13.920	3.804	4.570	2.581
75% (3rd quartile)	429.000	20.370	6.375	7.449	4.800
Maximum	656.000	58.780	34.470	18.077	22.228

Saturated fatty acid (SFA), monounsaturated fatty acid (MUFA), and polyunsaturated fatty acid (PUFA).

**Table 3:** Summary of predicted models for fatty acid classes in snacks.

Model	MSE			RMSE			MAE			R^2^		
	SFA	MUFA	PUFA	SFA	MUFA	PUFA	SFA	MUFA	PUFA	SFA	MUFA	PUFA
XGR	6.710	3.432	3.762	2.663	1.818	2.146	1.629	0.985	1.340	0.724	0.722	0.695
DT	10.735	5.120	7.075	3.303	2.194	2.680	1.594	1.124	1.259	0.418	0.514	0.390
LightGBM	7.440	3.401	4.434	2.668	1.837	2.155	1.582	1.153	1.303	0.722	0.742	0.635
LiR	9.086	3.689	5.820	3.010	1.919	2.409	2.031	1.354	1.684	0.510	0.662	0.503
RF	6.788	2.934	4.322	2.601	1.743	2.064	1.489	0.930	1.222	0.732	0.751	0.686

Mean squared error (MSE), root mean squared error (RMSE), mean absolute error (MAE), and coefficient of determination (R^2^), of extreme gradient boost (XGR) for decision tree (DT), light gradient-boosting machine (LightGBM), linear regression (LiR) and random forest (RF) models for predicting saturated fatty acid (SFA), monounsaturated fatty acid (MUFA), and polyunsaturated fatty acid (PUFA) content.

## Data Availability

Data for this study can be found at—https://wwwn.cdc.gov/nchs/nhanes/search/datapage.aspx?Component=Dietary&Cycle=2017-2018, accessed on 25 February 2022.
